# Supplementary material for: Assessment of Genetic Diversity of the “Acquaviva Red Onion” (Allium cepa L.) Apulian Landrace
Source: Plants (Basel). 2020 Feb 18;9(2):260. doi: 10.3390/plants9020260 (PMC7076509; doi:10.3390/plants9020260)
Supplement: Supplementary file 1 [file plants-09-00260-s001.pdf]

plants

Assessment of Genetic Diversity of the 'Acquaviva red onion' (*Allium cepa* L.) Apulian Landrace

Supplementary Material

plants MDPI

Table S1. Morphological descriptors related to bulb†, skin and flesh.

Code	Bulb shape (1: elliptic; 2: ovate; 3: broad elliptic; 4: globe; 5: broad ovate; 6: broad obovate; 7: rhomboid; 8: flat globe 9: flat)	Bulb apical shape (1: depressed; 2: flat; 3: weak prominent; 4: smooth; 5: weak pointed; 6: strong pointed)	Bulb basal shape (1: depressed; 2: pointed; 3: smooth; 4: weak conical; 5: strong conical)	Bulb thickness (cm)	CV* (%)	Bulb width (cm)	CV (%)	Thickness/Width	CV* (%)	Bulb skin colour (1: white; 2: gray; 3: green; 4: yellow; 5: brown: 6: dark pink; 7: violet-red	Skin colour intensity (3: weak; 5: intermediate; 7: dark)	Bulb flesh colour (1: white-light pink; 2: dark pink-violet; 3: violet- red)
ARO1	8	2	2	4,5 C	18,6	9,2 A	24,3	0,5 C	14,5	6	7	2
ARO2	8	2	2	5,1 C	18,6	10,0 A	16,8	0,5 C	29,4	6	7	2
ARO3	8	2	2	5,1 C	17,0	10,3 A	23,3	0,5 C	17,0	6	7	2
ARO4	8	2	2	4,6 C	15,9	9,8 A	16,8	0,5 C	13,0	6	7	2
ARO5	8	2	2	5,1 C	14,0	9,6 A	18,7	0,5 C	27,7	6	7	2
ARO6	8	2	2	4,4 C	9,6	9,1 A	19,1	0,5 C	27,7	6	7	2
ARO7	8	2	2	5,5 C	15,9	9,6 A	19,8	0,6 C	18,2	6	7	2
ARO8	8	2	2	4,9 C	15,6	10,9 A	17,3	0,5 C	19,2	6	7	2
ARO9	8	2	2	5,0 C	14,4	10,8 A	22,7	0,5 C	17,5	6	7	2
ARO10	8	2	2	4,9 C	20,2	9,8 A	21,3	0,5 C	18,0	6	7	2
ARO11	8	2	2	5,4 C	17,7	11,9 A	17,2	0,5 C	14,6	6	7	2
ARO12	8	2	2	5,3 C	14,6	10,3 A	19,2	0,5 C	34,8	6	7	2
ARO13	8	2	2	4,4 C	18,1	7,5 AB	24,0	0,6 C	15,2	6	7	2
MCO	6	3	4	7,6 B	14,0	6,8 B	24,2	1,1 B	22,3	5	5	1
TRO1	8	2	2	4,8 C	17,9	8,9 A	19,8	0,5 C	16,7	7	7	3
TRO2	7	6	6	11,6 A	20,6	6,6 B	20,7	1,7 A	18,3	7	7	3

†Means with the same letters in uppercase or lowercase are not statistically different at 0,01P or 0.05P, respectively (SNK's Test); * Coefficient of variation.

Table S2. Mean observed (Ho) and expected (He) heterozygosity and fixation index (F) for each population considered in this study.

ID Accession	Ho	He	F
**ARO1**	**0.40**	**0.45**	0.154
**ARO2**	0.44	0.52	0.184
**ARO3**	0.50	0.51	0.037
**ARO4**	0.48	0.50	0.050
**ARO5**	0.45	0.51	0.125
**ARO6**	0.55	0.50	-0.106
**ARO7**	0.27	0.28	0.063
**ARO8**	0.53	0.54	0.011
**ARO9**	0.44	0.48	0.085
**ARO10**	0.57	0.53	-0.040
**ARO11**	0.45	0.51	0.103
**ARO12**	0.39	0.46	0.109
**ARO13**	0.45	0.53	0.149
**TRO1**	0.53	0.50	-0.068
**TRO2**	0.46	0.45	0.013
**MCO**	0.51	0.50	0.001
**Mean**	0.46	0.49	0.054

Table S3. Pairwise population PhiPT values among all populations genotyped in the present study. Probability values (P) are based on 999 permutations.

Pairwise Comparisons		PhiPT	P
Accession 1	Accession 2		
ARO7	TRO2	0.468	0.001
ARO7	TRO1	0.427	0.001
ARO12	TRO2	0.339	0.001
ARO7	MCO	0.325	0.001
ARO7	ARO11	0.323	0.001
ARO7	ARO12	0.314	0.001
ARO12	TRO1	0.292	0.001
ARO3	ARO7	0.275	0.001
ARO4	ARO7	0.274	0.001
ARO5	ARO7	0.266	0.001
ARO2	ARO7	0.266	0.001
ARO7	ARO13	0.252	0.001
ARO12	MCO	0.234	0.001
ARO7	ARO10	0.230	0.001
ARO1	MCO	0.229	0.001
ARO9	TRO2	0.227	0.001
TRO2	MCO	0.227	0.001
ARO6	ARO7	0.224	0.001
ARO1	ARO7	0.223	0.001
ARO7	ARO9	0.223	0.001
ARO1	TRO1	0.219	0.001

ARO9	TRO1	0.216	0.001
ARO1	TRO2	0.207	0.001
ARO9	MCO	0.200	0.001
ARO8	TRO1	0.197	0.001
TRO1	MCO	0.196	0.001
ARO7	ARO8	0.194	0.001
ARO3	ARO12	0.194	0.001
ARO8	TRO2	0.188	0.001
ARO8	MCO	0.186	0.001
ARO8	ARO12	0.175	0.001
ARO5	TRO1	0.174	0.001
ARO6	TRO2	0.173	0.001
ARO3	MCO	0.172	0.001
ARO10	ARO12	0.171	0.001
ARO9	ARO12	0.171	0.001
ARO5	ARO12	0.166	0.001
ARO1	ARO12	0.164	0.001
ARO4	ARO12	0.162	0.001
ARO11	MCO	0.159	0.001
ARO13	TRO2	0.157	0.001
ARO6	TRO1	0.156	0.001
ARO5	TRO2	0.154	0.001
ARO6	ARO12	0.149	0.001
ARO3	TRO1	0.145	0.001
ARO3	TRO2	0.145	0.001
ARO6	MCO	0.139	0.001
ARO11	ARO12	0.135	0.001
ARO5	MCO	0.134	0.001
ARO4	MCO	0.133	0.001
ARO4	TRO2	0.132	0.001
ARO10	TRO2	0.130	0.001
ARO2	TRO1	0.128	0.001
ARO12	ARO13	0.127	0.001
ARO2	TRO2	0.122	0.001
ARO4	TRO1	0.120	0.001
ARO2	MCO	0.116	0.001
ARO1	ARO13	0.116	0.001
ARO9	ARO13	0.115	0.001
ARO8	ARO11	0.115	0.001
ARO10	TRO1	0.113	0.001
ARO11	TRO2	0.111	0.001
ARO9	ARO11	0.111	0.001
ARO2	ARO12	0.107	0.001

ARO8	ARO13	0.102	0.001
ARO13	TRO1	0.100	0.001
ARO13	MCO	0.100	0.001
ARO6	ARO11	0.096	0.001
ARO11	TRO1	0.094	0.001
ARO1	ARO11	0.093	0.001
ARO10	MCO	0.089	0.001
ARO3	ARO9	0.084	0.001
ARO4	ARO8	0.081	0.001
ARO3	ARO13	0.074	0.001
ARO2	ARO3	0.074	0.001
TRO1	TRO2	0.070	0.001
ARO1	ARO3	0.067	0.001
ARO10	ARO11	0.065	0.001
ARO5	ARO13	0.063	0.001
ARO4	ARO9	0.087	0.002
ARO6	ARO9	0.086	0.002
ARO3	ARO8	0.081	0.002
ARO5	ARO8	0.069	0.002
ARO9	ARO10	0.064	0.002
ARO3	ARO11	0.060	0.002
ARO2	ARO6	0.059	0.002
ARO5	ARO11	0.055	0.002
ARO4	ARO11	0.049	0.002
ARO4	ARO13	0.048	0.002
ARO6	ARO13	0.086	0.003
ARO5	ARO9	0.061	0.004
ARO2	ARO9	0.059	0.004
ARO2	ARO13	0.044	0.006
ARO1	ARO6	0.059	0.007
ARO1	ARO2	0.044	0.009
ARO1	ARO10	0.048	0.010
ARO3	ARO10	0.047	0.010
ARO2	ARO8	0.043	0.010
ARO2	ARO11	0.035	0.012
ARO5	ARO6	0.057	0.014
ARO8	ARO9	0.047	0.016
ARO1	ARO5	0.044	0.017
ARO5	ARO10	0.037	0.017
ARO1	ARO4	0.042	0.023
ARO2	ARO4	0.030	0.032
ARO8	ARO10	0.034	0.039
ARO10	ARO13	0.025	0.039

ARO1	ARO8	0.033	0.047
ARO6	ARO10	0.034	0.052
ARO1	ARO9	0.028	0.054
ARO4	ARO6	0.033	0.055
ARO3	ARO6	0.039	0.059
ARO6	ARO8	0.029	0.081
ARO11	ARO13	0.025	0.082
ARO3	ARO5	0.019	0.108
ARO2	ARO5	0.020	0.112
ARO4	ARO5	0.017	0.150
ARO4	ARO10	0.014	0.174
ARO3	ARO4	0.004	0.366
ARO2	ARO10	0.002	0.441

Table S4. Features of the 16 EST-SSR and 21 genomic SSR primer pairs tested on the 16 populations of the present study.

Locus	Primer Sequences (5′→3′)	Repeat Motif	Annealing Temp. (°C)	Reference
**ACM006**	GCAGTTCTCCCTTTGTAAAATCA<br>GTGATGGATGAGTGGATGGA	$(CTC)_7$	58	Kuhl et al., 2004
**ACM024**	CCCCATTTTCTTCATTTTCTCA<br>TGCTGTTGCTGTTGTTGTTG	$(GCA)_{10}$	58	Kuhl et al., 2004
**ACM091***	TCTCCTCCTCTAACCAGCCA<br>GGTGCTCCAGTTGAGCTTTC	$(TCT)_{10}$	58	Kuhl et al., 2004
**ACM101***	CCTTTGCTAACCAAATCCGA<br>CTTGTTGAGAAGGAGGACGC	$(TCC)_5$	58	Kuhl et al., 2004
**ACM115**	TCCATCTATGCATCTGCCAC<br>CTATTCTTCCACTGGGGCAA	$(CAC)_6$	58	Kuhl et al., 2004
**ACM121**	GCAAACTCATATAGTGCCGC<br>GAACCGATTCTACGAGCAGC	$(TAT)_5$	58	Kuhl et al., 2004
**ACM132***	ATGGGGCCTGGTAAGTTTTT<br>TGCACACCGTTTCCATTTTA	$(ACAT)_{14}AC(CATG)_4$	58	Kuhl et al., 2004
**ACM134**	ACACACACAAGAGGGAAGGG<br>CACACACCCACACACATCAA	$(GA)_8$	58	Kuhl et al., 2004
**ACM138***	ACGGTTTGATGCACAAGATG<br>CCAACCAACAGTTGATACTGC	$(CTGC)_{11}$	58	Kuhl et al., 2004
**ACM147***	CACTTTCCCGTCTAATCGACA<br>TTCCCACAATCAAAACACCA	$(CTC)_5$	58	Kuhl et al., 2004
**ACM151**	TGTCAGACAAGCAACTCCTCC<br>AGGTGAGGCTTAGATGGGGT	$(ACA)_5$	58	Kuhl et al., 2004
**ACM152***	TCCCAAGAGTCCAAGAATGG<br>TGTTCTCCCTTAAACAGTGCAA	$(CAG)_8$	58	Kuhl et al., 2004
**ACM227**	AGCAGCTCATTCAGCAAAA<br>GAGGTCGGAGAAGGAGGAGT	$(CAG)_7$	58	Kuhl et al., 2004
**ACM235***	ACGCATTTTCAAATGAAGGC<br>TGAGTCGGCACTCACCTATG	$(TTTG)_4$	58	Kuhl et al., 2004
**ACM238**	TGATAGCCAGTTGATTGCGA<br>TTCCCCAGTACACACCTTCC	$(TTTG)_4$	58	Kuhl et al., 2004
**ACM303**	CGATGATTATAGATATTTCGTTATGG<br>CATTCATAATCTCACAGTGGAAGG	$(AC)_8$	58	Kuhl et al., 2004
**ACM359**	TCCATTTCACTTGGGTTTACAA<br>GCAAAGAATTAAATTGGAAGAAA	$(TATG)_5$	58	Baldwin et al., 2012
**ACM366**	TTTTGTCTAAAAATGGGCTGA	$(A)_{10}$	58	Baldwin et al.,

	TGGGCTAATTCAGGGTTG			2012
**ACM373**	AGGTTAAGAAGTTGAATGGTCTG<br>AAATGGACAAGTGGCATTCA	$(AC)_{15}$	58	Baldwin et al., 2012
**ACM376**	TCCAAAACCCATAGGTTCCA<br>CAAGTTGGAAGGATAAGTTGTCTG	$(AC)_{11}$	58	Baldwin et al., 2012
**ACM385**	AAGGATTTTATATTGATTTTGAATGAG<br>CCAATTTCAAAAGACAGCATTT	$(AATA)_5$	58	Baldwin et al., 2012
**ACM389**	TGGAAAATGGAGCATGGTT<br>GGTAGTAGAGGTGGGGCGTT	$(AAT)_5$	58	Baldwin et al., 2012
**ACM390**	ACATACAAGATGGACACTCAACA<br>GTCAACAACCATGAGTCCG	$(AT)_{10}(GTAT)_9$	58	Baldwin et al., 2012
**ACM410**	TGAGGCACGATGAAACCATA<br>TTTTTGTTCCCACTCATCCTTT	$(AT)_6$	58	Baldwin et al., 2012
**ACM439**	AAATGAAATACTGGAAAGAATAATTGA<br>ACCCTTAGCCATGAACCTCC	$(TA)_7$	58	Baldwin et al., 2012
**ACM443**	TGGTGCTTGCTATGTTTTGC<br>CCCTAGGCCAAGCTTACTTGT	$(TA)_{10}$	58	Baldwin et al., 2012
**ACM446***	TCAAGAATTCTGTTGCATCTTGT<br>AATAAGACCGCAGAAACGAAA	$(TA)_8$	65 ⟶ 55*	Baldwin et al., 2012
**ACM449***	GTAAAGGTGTAATAGGAATGAATCG<br>TACAAAGAAACACACGCGCT	$(GT)_7$	65 ⟶ 55*	Baldwin et al., 2012
**ACM452**	ATCGGGGGTTTGGAATAAAA<br>ACGTGATAGGGTGGCTGAAC	$(AT)_7$	58	Baldwin et al., 2012
**ACM463***	GCATATCGTTAATATTTCATACTTCCA<br>GCTTCGCAACAGACACGTTA	$(AC)_8$	58	Baldwin et al., 2012
**ACM474**	TACGGGGAATCAACAACTGC<br>TACGCCGTTAGTAAGCGGAT	$(AT)_6$	58	Baldwin et al., 2012
**ACM477**	TGCAATTGGAACTTTGGTTTT<br>CCGTTCCTCTATTTTGCAGC	$(AT)_6$	58	Baldwin et al., 2012
**ACM504***	TCCCAAGAGTCCAAGAATGG<br>GTCGGTTTCAACATGAATACCAT	$(GT)_6$	58	Baldwin et al., 2012
**ACM509**	CAATAATTAGCGGAAACAAAGAA<br>TCACCTTTGGAGTTTGGAGC	$(TA)_6$	58	Baldwin et al., 2012
**ACM520**	CATGAAGCTGTTATTTGCATTTG<br>CCCTGCATGATGTTATTTTGC	$(TA)_7$	58	Baldwin et al., 2012
**ACM531**	GTACCCGAGACAAACCCCTT<br>GAAGTTGCTGGGAGAGATGC	$(TC)_6$ACCCCATCTTCTTCTCCTCTTCCTGTTTCTTCTTCTCACTTTA$(T)_{10}$	58	Baldwin et al., 2012
**Gssr6**	CAAGAGGCCAATCATGTGATAA<br>AGGCTTTGATGCTGTTTTTGAT	(TAT)4	58	Kim et al., 2012

* Primer pairs selected to genotype the 16 populations used in this study.

Table S5. Pairwise population matrix of Nei genetic distance among the 16 onion populations characterized in the present study.

ARO1	ARO2	ARO3	ARO4	ARO5	ARO6	ARO7	ARO8	ARO9	ARO10	ARO11	ARO12	ARO13	TRO1	TRO2	MCO	
0.000																**ARO1**
0.041	0,000															**ARO2**
0,051	0.078	0.000														**ARO3**
0.034	0.040	0.043	0.000													**ARO4**
0.046	0.046	0.053	0.051	0.000												**ARO5**
0.058	0.070	0.060	0.042	0.069	0.000											**ARO6**
0.230	0.274	0.270	0.253	0.266	0.237	0.000										**ARO7**
0.052	0.040	0.073	0.068	0.067	0.054	0.233	0.000									**ARO8**
0.022	0.042	0.034	0.036	0.033	0.043	0.232	0.035	0.000								**ARO9**
0.062	0.031	0.068	0.028	0.065	0.076	0.250	0.052	0.055	0.000							**ARO10**
0.050	0.060	0.037	0.046	0.063	0.072	0.288	0.072	0.039	0.066	0.000						**ARO11**
0.109	0.119	0.169	0.140	0.158	0.106	0.253	0.123	0.121	0.166	0.128	0.000					**ARO12**
0.087	0.072	0.086	0.064	0.084	0.094	0.221	0.081	0.067	0.046	0.049	0.125	0.000				**ARO13**
0.159	0.131	0.118	0.095	0.167	0.125	0.461	0.151	0.138	0.102	0.090	0.273	0.106	0.000			**TRO1**
0.151	0.114	0.115	0.110	0.142	0.151	0.573	0.138	0.138	0.116	0.097	0.330	0.150	0.062	0.000		**TRO2**
0.156	0.113	0.133	0.102	0.122	0.101	0.252	0.125	0.111	0.072	0.145	0.206	0.103	0.159	0.179	0.000	**MCO**

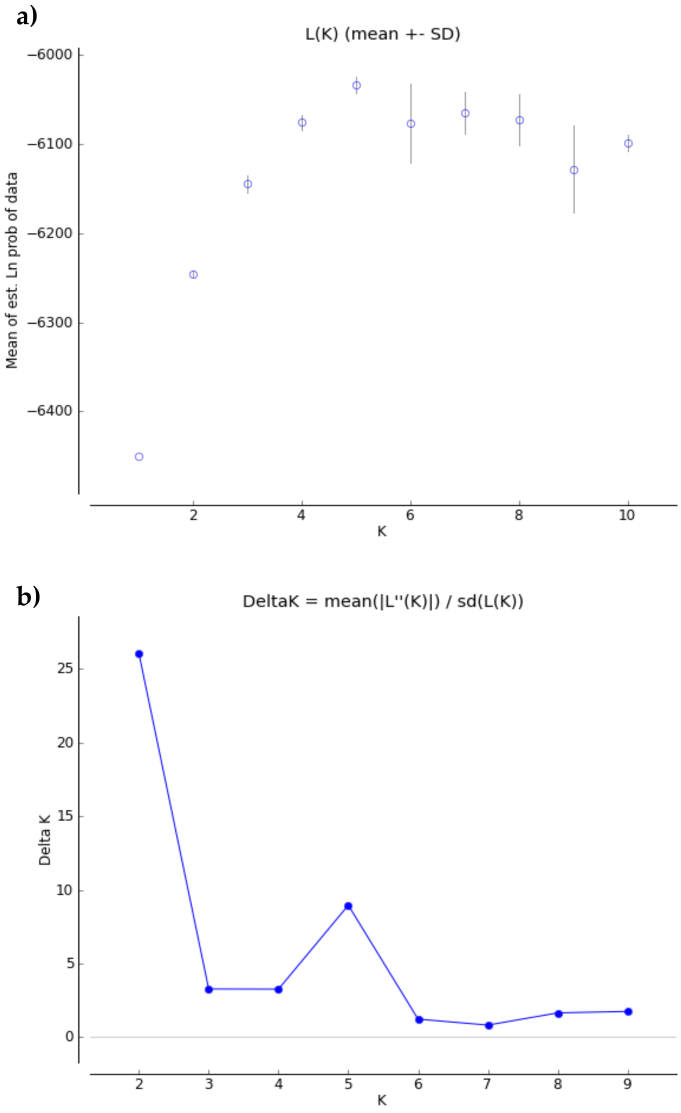

Figure S1. Post-hoc evaluation of STRUCTURE results. Mean likelihood L(K) and variance for 10 independent runs in each of the hypothetical assumed populations (1-10) (**a**) and detection of the most likely value of K (**b**). Two populations best define genetic variation in *Allium cepa* collection. although the next highest peak describes a deeper structuring in the collection. as confirmed both by the hierarchical clustering and by the principal coordinate analysis**.**
